# Characterization of Fibrous Protein Gel Network Properties in Brewer’s Yeast-Enhanced Meat Analogs Produced by High-Moisture Extrusion

**DOI:** 10.3390/gels12090769

**Published:** 2026-08-27

**Authors:** Yu Zhang, Yung-Hee Jeon, Ayeon Han, Gi-Hyung Ryu, Bon-Jae Gu, Da-Eun Jung

**Affiliations:** 1 Department of Food and Quality Engineering, Nanning University, Nanning 530200, China; 2 Department of Food Science and Technology, Food and Feed Extrusion Research Center, Kongju National University, Yesan 32439, Republic of Korea; 3 Department of Food and Resource Economics, Dankook University, Cheonan 31116, Republic of Korea

**Keywords:** *Saccharomyces cerevisiae*, meat substitutes, plant proteins, soybean proteins, glutens, gels

## Abstract

The development of structured protein gel matrices with meat-like fibrous properties is a key challenge in the design of high-moisture meat analogs (HMMA). This study incorporated brewer’s yeast into a soy protein-wheat gluten-corn starch matrix and evaluated the stage-specific effects of moisture content (MC), barrel temperature (BT), and screw speed (SS) on the fibrous appearance, integrity index, nitrogen solubility index (NSI), texture, and cutting strength of yeast-enhanced meat analogs (Y-MA), alongside pasting, rheological, Fourier transform infrared spectroscopy (FTIR), and Raman analyses of pre-extrusion blends (0% and 10% yeast). Among the individual factors examined under their respective fixed processing conditions, moisture content produced the largest changes in several measured responses. Raising MC from 55% to 70% weakened the fibrous structure, reduced chewiness from 6211 to 867 g, and lowered cutting strength in both directions. Raising BT from 140 to 170 °C produced more distinct fibrous features, higher NSI, and greater firmness. Increasing SS from 150 to 300 rpm was associated with decreased chewiness. The 10% yeast blend showed lower pasting viscosities, a higher pasting temperature, and lower terminal G′ and G″ than the yeast-free blend, while FTIR and Raman spectra remained unchanged. These findings indicate that extrusion parameters influenced the fibrous appearance, matrix retention, and mechanical properties of Y-MA under the fixed conditions tested.

## 1. Introduction

Plant-based meat analogs have attracted increasing attention from the food industry and consumers as alternatives to conventional meat products [1]. The demand for plant-based meat analogs has increased in recent years in response to environmental, health, and ethical concerns, together with growing interest in vegetarian and plant-oriented diets [2,3]. From a structural perspective, successful product development depends largely on the formation of protein-based matrices capable of providing meat-like texture, juiciness, and fibrous architecture. Accordingly, advanced processing technologies, including extrusion, shear-cell processing, and three-dimensional printing, are used to restructure plant proteins and produce anisotropic fibrous matrices [2,3,4]. Soy, wheat gluten, pea, and mung bean proteins are commonly used as major protein sources, while plant-based fats, flavoring agents, colorants, and other functional ingredients are incorporated to improve texture, juiciness, flavor, and appearance [2,3]. On a dry matter basis, yeast typically contains approximately 40 to 60% crude protein [5], making it a promising protein-rich ingredient for structured food systems. In the brewing industry, yeast is generated as a major by-product and is often discarded or underutilized. However, brewer’s yeast is a natural source of vitamins, especially B-complex vitamins, and provides essential minerals and antioxidants [6].

Brewer’s spent yeast also contains moderate amounts of ash and relatively low lipid levels, while its amino acid profile generally includes substantial amounts of glutamic and aspartic acids, together with essential amino acids such as leucine, lysine, threonine, and valine [7,8]. Unlike soy protein isolate, which is a highly purified protein ingredient, brewer’s yeast is a heterogeneous cellular material containing yeast proteins together with cell-wall-derived polysaccharides and other non-protein constituents [6,9]. These compositional differences may influence hydration, water binding, melt rheology, phase distribution, and the continuity of the soy protein-wheat gluten matrix during high-moisture extrusion. Previous studies have reported that the incorporation of brewer’s yeast or yeast-derived protein can alter the fibrous morphology and textural properties of soy-based high-moisture extrudates [10,11]. Therefore, brewer’s yeast was expected to modify the physical and structuring responses of the soy protein-wheat gluten matrix to extrusion conditions.

Extrusion cooking is one of the major industrial texturization technologies used to manufacture plant-based meat analogs [1,12]. This process involves the simultaneous application of heat, mechanical energy, pressure, and shear forces to a dough-like protein mixture, transforming it into a structured product with desirable texture, structure, and nutritional properties [13,14,15]. In high-moisture extrusion, these thermal and mechanical inputs promote protein denaturation, unfolding, molecular rearrangement, aggregation, and alignment. When followed by cooling in the die, these changes can stabilize a fibrous and anisotropic gel-like protein matrix. Thus, high-moisture extrusion can be regarded as a dynamic structuring process that converts hydrated protein blends into fibrous protein gel networks.

The effects of processing parameters on meat analogs are multifaceted, involving thermal, mechanical, and compositional factors that interact to determine the final texture, structure, and gel network characteristics. The rheological and cooking characteristics of meat analog dough are primarily dependent on its moisture level [16,17,18]. Appropriate moisture content is necessary to facilitate protein hydration, regulate melt viscosity, promote starch plasticization, and ensure proper material flow during extrusion [19]. Moisture content also affects protein denaturation and aggregation in high-moisture-extruded mung bean protein isolate, with optimal quality, including high solubility and strong gel formation behavior, observed at 49.3% moisture content [20]. Barrel temperature plays a critical role in the denaturation, unfolding, and rearrangement of proteins, thereby influencing the structural integrity and mechanical properties of the resulting gel matrix. An increase in temperature can induce protein unfolding and aggregation, which may strengthen protein–protein interactions and improve structural firmness [21,22]. Nor Afizah and Rizvi reported that texturized whey protein concentrate extruded at 50 and 70 °C formed soft structured aggregates with high solubility, whereas extrusion at 90 °C produced protein aggregates with low solubility [23]. Screw speed is also associated with residence time, shear intensity, mixing, protein restructuring, and fiber alignment during extrusion [18]. Therefore, controlling moisture content, barrel temperature, and screw speed is essential for regulating extrusion-induced protein gel network formation and the mechanical performance of high-moisture meat analogs (HMMA).

Although previous studies have extensively investigated the effects of extrusion parameters on conventional plant-based meat analogs based on soy, pea, or wheat proteins [24,25], only a limited number of studies have examined formulations incorporating brewer’s yeast or other brewery by-products [10,11]. These studies have primarily focused on ingredient incorporation, nutritional enhancement, or general product quality, rather than systematically evaluating the stage-specific effects of key high-moisture extrusion parameters on the macroscopic fibrous appearance, assay-defined matrix retention, nitrogen extractability, and mechanical properties of brewer’s yeast-containing systems [10,11]. Therefore, the novelty of the study lies in the sequential, one-factor-at-a-time (OFAT) evaluation of the individual effects of moisture content, barrel temperature, and screw speed on these properties, together with complementary Rapid Visco Analysis (RVA), rheological, Fourier transform infrared spectroscopy (FTIR), and Raman characterization of the pre-extrusion blends. This approach was used to identify the individual effects of each processing variable under the corresponding fixed conditions at each stage, providing a basis for factorial or multivariable optimization studies.

The objectives were to: (1) evaluate the effects of each processing parameter on macroscopic fibrous appearance, assay-defined matrix retention, nitrogen extractability, and mechanical properties; (2) characterize the pasting, thermal viscoelastic, FTIR, and Raman profiles of pre-extrusion blends; and (3) identify, within the tested range of each variable, the processing levels associated with comparatively favorable structural and mechanical responses, without implying an optimized combination across variables.

## 2. Results and Discussion

### 2.1. Pasting Properties of Pre-Extrusion Blends

**Figure 1:**
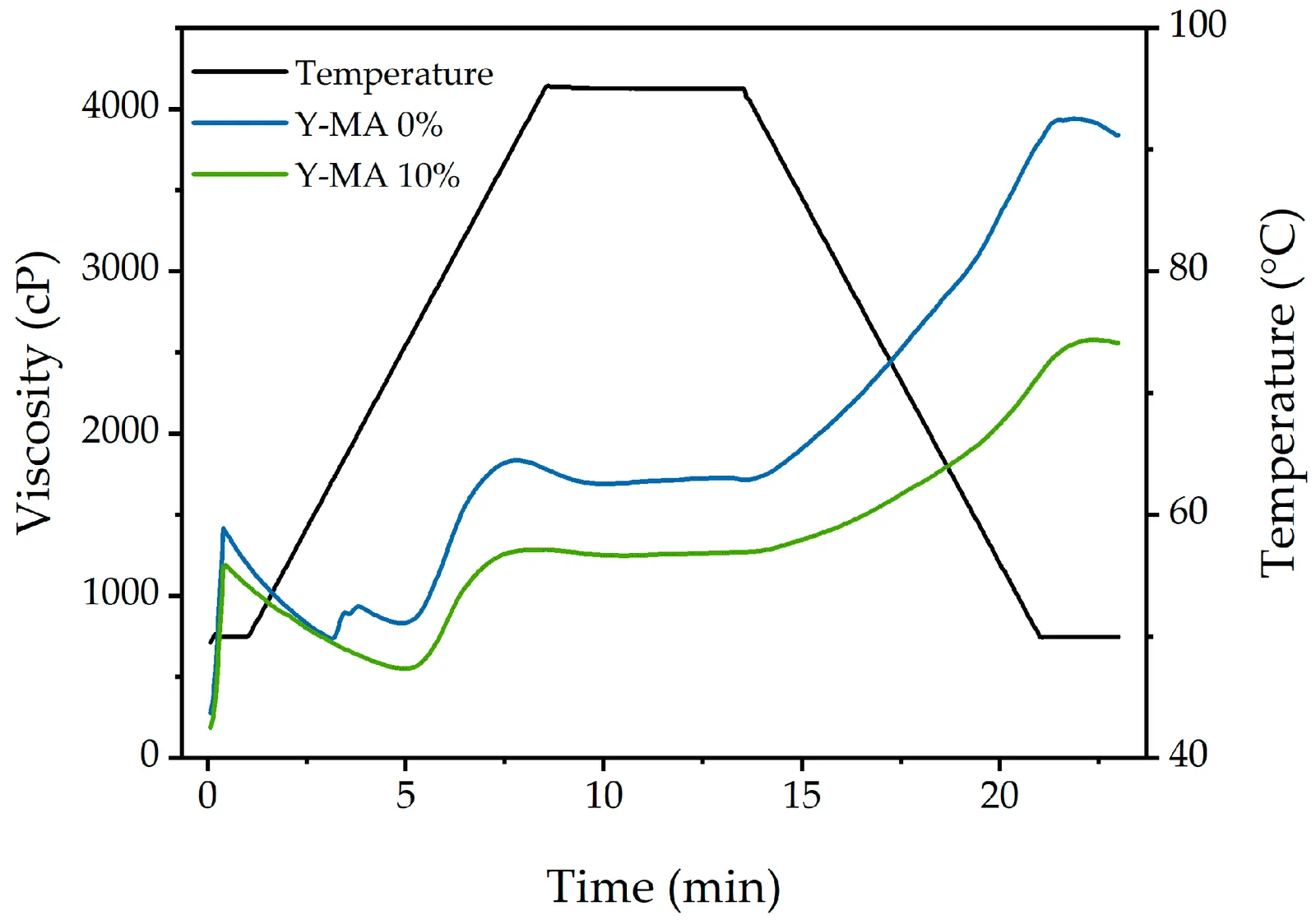
RVA pasting profiles of pre-extrusion blends containing 0% and 10% brewer’s yeast.

The pasting properties of the pre-extrusion blends are shown in Figure 1. Throughout the RVA heating and cooling cycle, the 10% Y-MA blend exhibited a lower viscosity profile than the 0% Y-MA blend. The lower peak and trough viscosities indicate that the yeast-containing formulation developed less viscosity during heating, which may reflect restricted starch granule swelling and amylose leaching. Because brewer’s yeast was incorporated by replacing part of the original soy protein isolate-wheat gluten-corn starch blend, the corn starch content decreased from 10% in Y-MA 0% to 9% in Y-MA 10%. Therefore, the lower viscosity should be interpreted as the combined effect of brewer’s yeast incorporation and changes in the overall formulation rather than solely as an effect of individual yeast components.

Breakdown viscosity decreased substantially from 123.00 ± 34.60 cP in Y-MA 0% to 18.00 ± 2.00 cP in Y-MA 10%. Although a lower breakdown viscosity is often associated with greater resistance to continued heating and mechanical shear, the markedly lower peak viscosity of Y-MA 10% indicates that this reduction may primarily reflect limited initial swelling and a smaller viscosity increase rather than unequivocally improved paste stability. During cooling, Y-MA 10% also showed lower final and setback viscosities. Final viscosity decreased from 3838.67 ± 212.26 to 2559.67 ± 64.50 cP, while setback viscosity decreased from 2124.33 ± 147.28 to 1293.33 ± 28.29 cP. These reductions suggest that the brewer’s yeast-containing blend underwent less viscosity development during cooling and may have exhibited reduced reassociation of gelatinized starch components under the applied RVA conditions.

In contrast to the decreases in viscosity parameters, the RVA-derived pasting temperature increased from 50.20 ± 0.35 °C in Y-MA 0% to 76.48 ± 0.23 °C in Y-MA 10%. This result indicates that the onset of measurable viscosity development was delayed in the brewer’s yeast-containing formulation. One possible explanation is that hydration of yeast-derived proteins and cell-wall polysaccharides altered water distribution within the blend and reduced the water readily available for starch swelling. Interactions among starch, proteins, and yeast-derived polysaccharides may also have restricted granule expansion during heating [26,27]. However, β-glucan content and water distribution were not directly measured; therefore, these mechanisms remain tentative.

Previous studies have demonstrated that the influence of brewer-derived ingredients on starch pasting behavior depends on the composition and processing history of the added material. Satrapai and Suphantharika [28] reported increased pasting viscosities when isolated brewer’s yeast β-glucan was incorporated into rice starch, whereas reduced viscosity development has been observed in cereal systems containing brewer-derived fiber-rich ingredients [29]. The difference between these findings and the present results may be associated with variations in starch source, β-glucan purity and molecular properties, protein content, ingredient concentration, and prior processing conditions. Overall, the RVA results demonstrate that the incorporation of 10% brewer’s yeast substantially altered the hydration and pasting behavior of the pre-extrusion blend.

### 2.2. Rheological Properties

**Figure 2:**
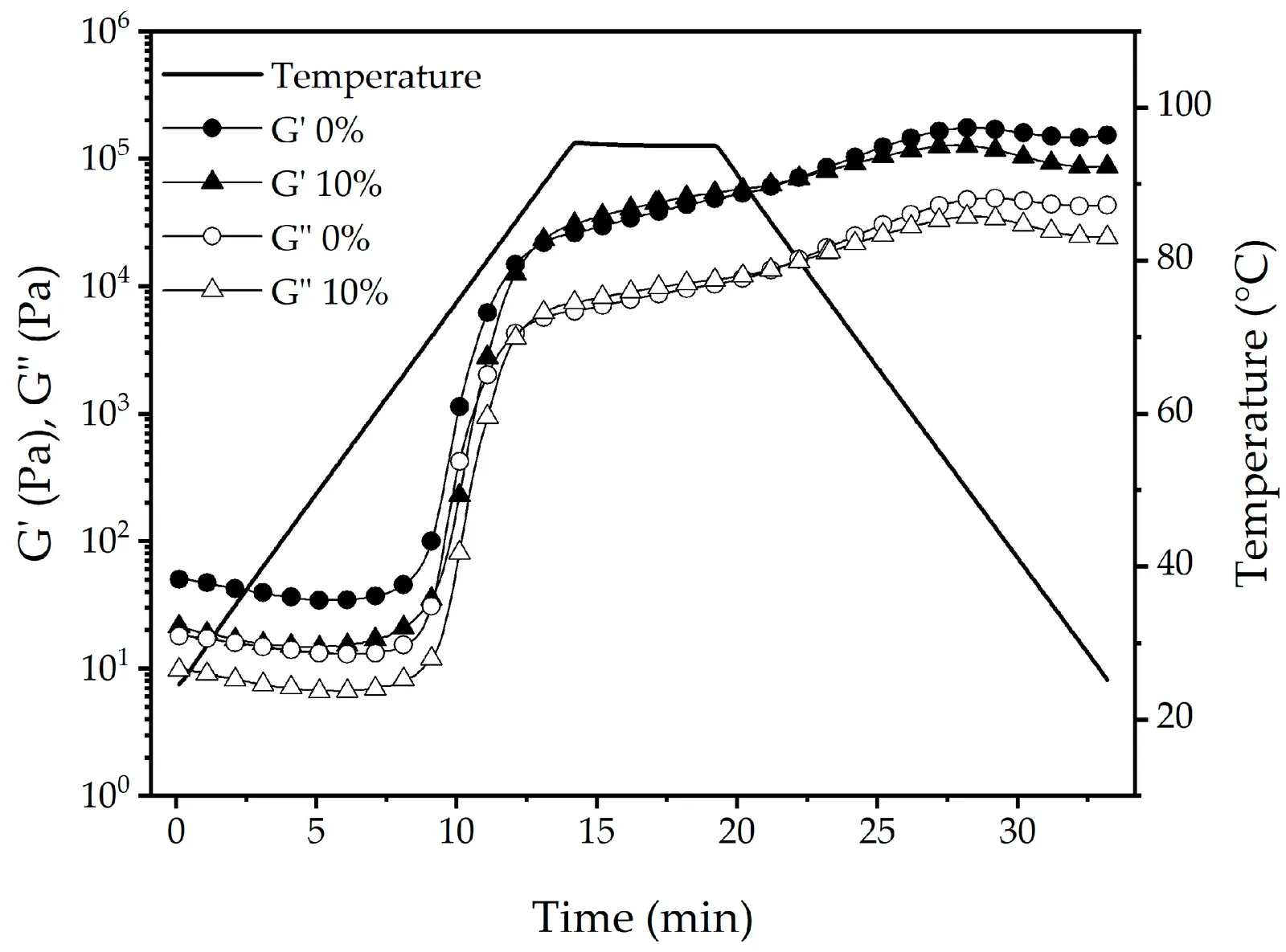
Temperature-dependent changes in storage modulus (G′) and loss modulus (G″) of pre-extrusion blends with and without brewer’s yeast during temperature sweep measurements.

The temperature-dependent rheological properties of the pre-extrusion blends are presented in Figure 2. At the initial stage, both blends exhibited higher G′ than G″, indicating predominantly elastic behavior before heating. Upon heating, G′ and G″ increased sharply at approximately 9–12 min, reflecting the thermal development and strengthening of the protein-starch composite network. Protein-starch systems can behave as composite gels in which starch gelatinization and thermally induced protein aggregation jointly contribute to the increase in viscoelastic moduli during heating [30]. During the high-temperature holding stage, the moduli of the 10% yeast blend approached or temporarily exceeded those of the control, indicating that brewer’s yeast did not uniformly reduce G′ and G″ throughout the temperature sweep. During cooling, both moduli increased further; however, the control exhibited a greater increase and higher terminal G′ and G″ values than the 10% yeast blend. This result suggests that brewer’s yeast primarily limited the final consolidation of the thermally developed network during cooling. The lower terminal moduli may reflect the combined effects of dilution of the soy protein-wheat gluten-starch matrix, altered water distribution, and interference by yeast-derived proteins and cell-wall polysaccharides, rather than the effect of β-glucan alone. Previous studies have similarly demonstrated that spent brewer’s yeast β-glucan can modify starch gelatinization and gel development, although the direction and magnitude of these effects depend on the starch source, β-glucan structure, concentration, and formulation [28,31,32]. Throughout the test, G′ remained higher than G″ without crossover, confirming that both blends maintained elastic-dominant viscoelastic behavior. Recent studies have linked the viscoelastic properties of plant-protein mixtures measured under extrusion-relevant thermal conditions with the mechanical and anisotropic characteristics of the resulting high-moisture extrudates [33].

### 2.3. Fourier Transform Infrared Spectroscopy (FTIR)

**Figure 3:**
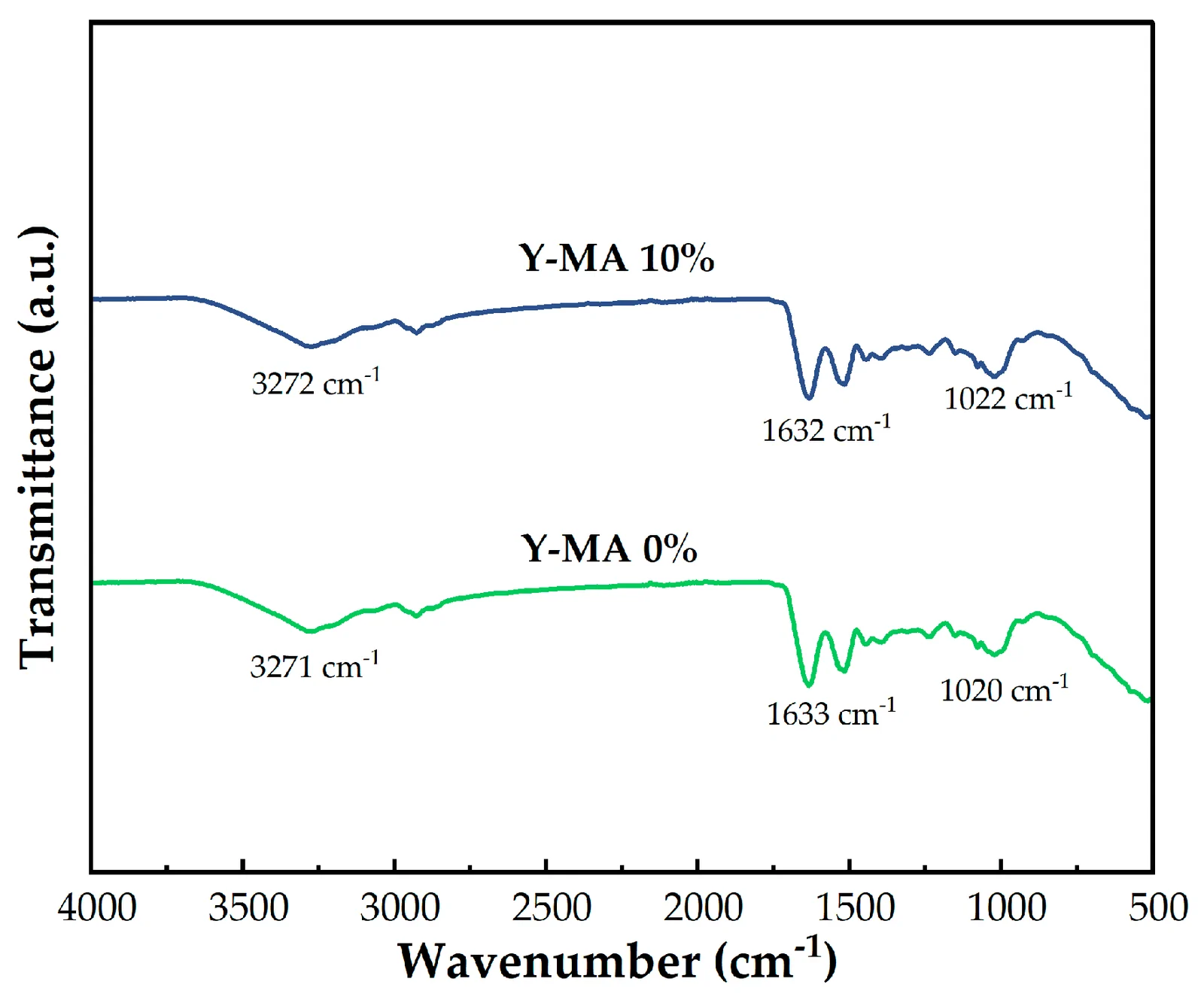
FTIR spectra of the pre-extrusion blends containing 0% and 10% brewer’s yeast.

The FTIR spectra of the pre-extrusion blends are shown in Figure 3. Both samples exhibited similar overall spectral profiles, with broad bands at 3272 and 3271 cm^−1^, bands at 1632 and 1633 cm^−1^, and carbohydrate-associated bands at 1022 and 1020 cm^−1^ for Y-MA 0% and Y-MA 10%, respectively. The broad band near 3270 cm^−1^ was attributed to overlapping O-H and N-H stretching vibrations, whereas the band near 1632–1633 cm^−1^ may include contributions from protein amide I vibrations and the H-O-H bending vibration of bound water. The band near 1020 cm^−1^ was associated with the carbohydrate fingerprint region, including C-O and C-O-C vibrations originating from starch and yeast-derived polysaccharides [32,34,35]. The differences in peak positions between the two blends were limited to 1–2 cm^−1^, which was smaller than the spectral resolution of 4 cm^−1^, and no additional resolved absorption bands were observed. Therefore, brewer’s yeast addition did not produce a detectable alteration in the dominant functional-group profile of the pre-extrusion blend [35]. When considered together with the rheological results, the FTIR findings indicate that the reduction in terminal viscoelastic moduli occurred without major FTIR-detectable changes in the bulk chemical profile. This combination may be consistent with changes in physical network organization and water distribution within the protein-starch composite matrix [30,36].

### 2.4. Raman Spectroscopy

**Figure 4:**
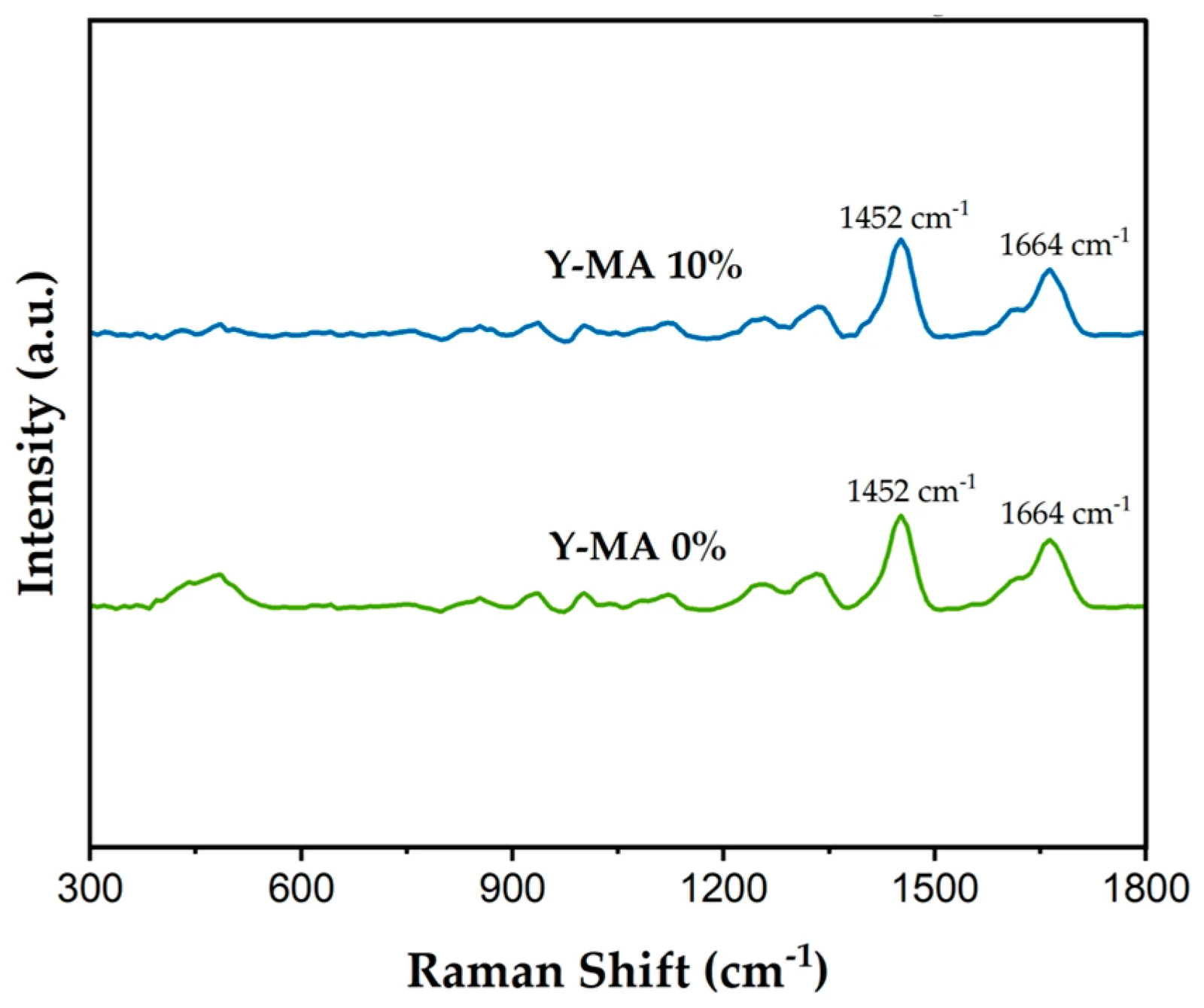
Raman spectra of pre-extrusion blends with and without brewer’s yeast over the Raman shift range of 300–1800 cm^−1^.

The Raman spectra of the pre-extrusion blends are presented in Figure 4. Both Y-MA 0% and Y-MA 10% exhibited similar overall spectral profiles, with prominent bands at 1452 and 1664 cm^−1^. The band at 1452 cm^−1^ was assigned to CH_2_ and CH_3_ bending vibrations associated mainly with aliphatic molecular groups, whereas the band at 1664 cm^−1^ corresponded to the amide I region, which is dominated by C=O stretching vibrations of the protein peptide backbone [37,38]. Similar protein-associated bands at 1452 and 1664 cm^−1^ have also been identified in Raman spectra of *Saccharomyces cerevisiae* cells [38]. No discernible shifts in these major bands or additional resolved Raman bands were observed following brewer’s yeast addition, indicating that the incorporation of brewer’s yeast did not produce a detectable alteration in the dominant Raman-active vibrational profile of the pre-extrusion blend [39]. When considered together with the FTIR and rheological results, the Raman findings indicate that the lower terminal viscoelastic moduli of Y-MA 10% occurred without marked shifts in the principal spectroscopic bands. This combination may be consistent with changes in hydration, dilution of the original soy protein-wheat gluten-starch matrix, or physical organization of the composite network rather than the formation of new Raman-detectable chemical structures [35].

### 2.5. Fibrous Protein Gel Network Formation

**Figure 5:**
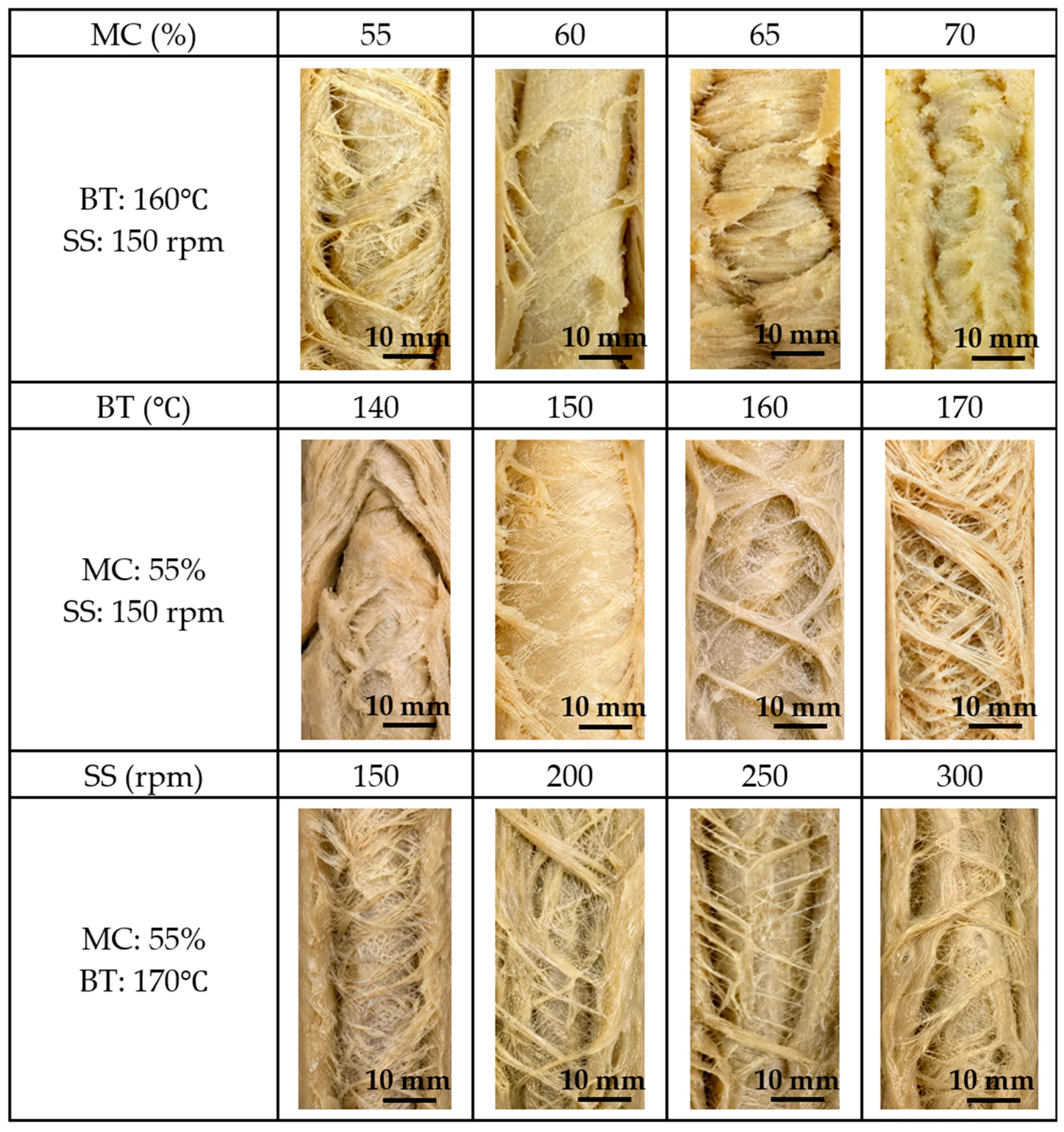
Macroscopic fibrous morphology of Y-MA produced under different high-moisture extrusion conditions. Scale bars represent 10 mm.

The fibrous structure of Y-MA is shown in Figure 5. Fibrous structure is commonly regarded as a morphological feature associated with anisotropic structuring in HMMA. During high-moisture extrusion, thermal and shear treatments may induce protein denaturation and expose previously buried functional groups. The resulting protein chains may subsequently undergo alignment and aggregation through covalent and non-covalent interactions, including disulfide bonding, hydrogen bonding, and hydrophobic interactions, which have been proposed to contribute to the development of fibrous, gel-like matrices during cooling in the die [14,40].

Moreover, a long cooling die is crucial for suppressing excessive expansion by preventing overheating and moisture evaporation, thereby stabilizing the aligned protein gel network [41,42]. Current models propose that anisotropic morphology develops through the deformation and alignment of phase-separated domains, followed by structural fixation as viscosity increases during cooling in the die. NMR and MRI observations of soy protein extrudates have identified phase-separated lamellar regions whose thickness increased during passage through the cooling die [43]. Figure 5 illustrates the fibrous structures of Y-MA produced under different processing conditions, including moisture content, barrel temperature, and screw speed. As moisture content increased, the fibrous gel network of Y-MA became progressively weaker. The sample produced at 55% moisture content exhibited a dense and well-developed fibrous structure, whereas the sample produced at 70% moisture content showed almost no visible fibrous network. This result suggests that excessive moisture may weaken protein–protein interactions and reduce the mechanical stress required for fiber alignment during extrusion. Similarly, it has been reported that HMMA formulated at 55% moisture content forms a more distinct fibrous structure than samples produced at 60% moisture content [18].

A tendency toward more distinct fibrous morphology was observed as barrel temperature increased. In particular, Y-MA produced at 170 °C showed the most pronounced macroscopic fibrous features among the tested samples. This observation may reflect temperature-dependent differences in protein alignment and restructuring during extrusion and cooling. In contrast, screw speed did not markedly affect the visible fibrous structure of Y-MA. Under the selected moisture and barrel temperature conditions, Y-MA produced at all four screw speeds maintained dense and abundant fibrous structures. Samard reported similar results, indicating that screw speed had no significant effect on the fiber structure of HMMA [44]. Taken together, moisture content and barrel temperature were more closely associated with differences in macroscopic fibrous morphology than screw speed under the tested conditions.

### 2.6. Integrity Index

**Figure 6:**
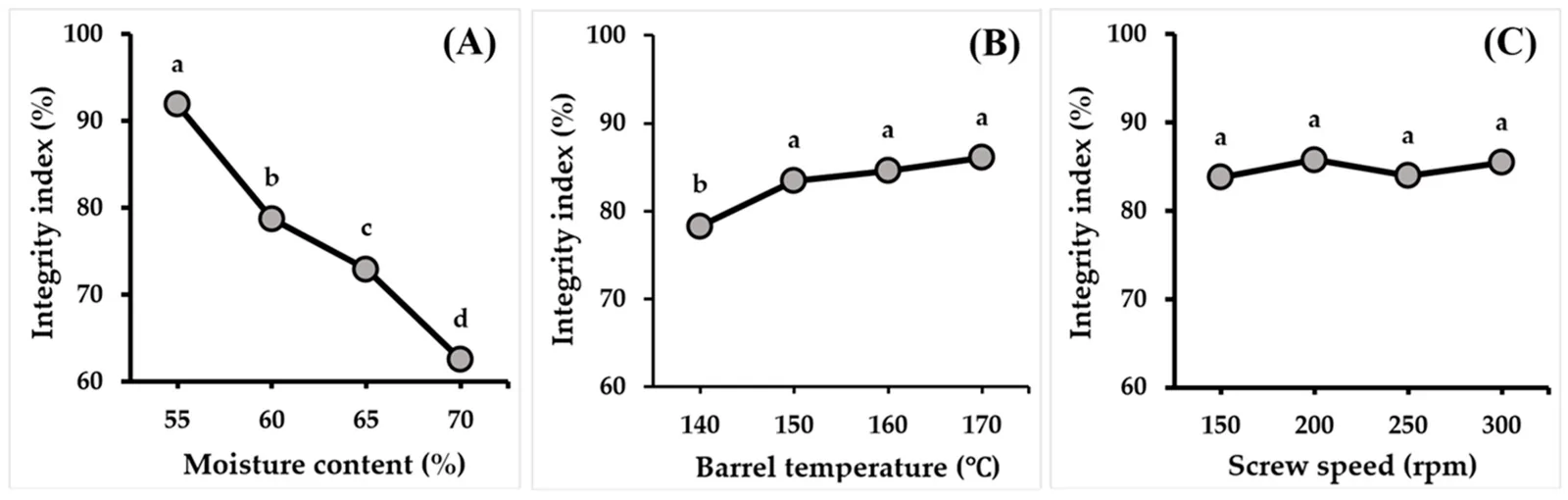
Integrity index of Y-MA produced under different high-moisture extrusion conditions: (**A**) moisture content, (**B**) barrel temperature, and (**C**) screw speed. Data are presented as mean ± SD (n = 3). Different lowercase letters indicate significant differences among treatment levels within each processing parameter (*p* < 0.05).

The integrity index is presented in Figure 6. The integrity index represents the percentage of dry material retained after autoclaving, homogenization, washing, filtration, and drying [18,44]. Accordingly, a higher value indicates greater relative matrix retention and lower material loss under the specified assay conditions. Moreover, because the assay involved autoclaving followed by homogenization and washing, the resulting values represent resistance to a standardized severe challenge. The integrity index of Y-MA under various extrusion operating conditions is shown in Figure 6. Water plays a dual role during high-moisture extrusion. At lower moisture levels, water may act primarily as a plasticizer, reducing melt viscosity and facilitating protein molecular mobility and rearrangement without excessively disrupting protein–protein interactions. This condition may promote the formation of a cohesive and structurally stable gel network, as reflected by the highest integrity index observed at 55% moisture content.

From a sensory perspective, a higher integrity index may indicate a cohesive and structurally stable matrix that contributes to meat-like textural attributes, including firmness, chewiness, and fibrousness. Previous studies have demonstrated associations between instrumental mechanical properties and sensory perceptions of chewiness, hardness, and structural compactness in plant-based meat analogs [45]. However, a higher integrity index should not be interpreted as a direct predictor of consumer acceptance, because sensory preference depends on the balance among firmness, tenderness, juiciness, flavor, and other product-specific attributes. Excessive hardness or chewiness may negatively affect mouthfeel and product pleasantness [46].

As moisture content increased beyond 60%, the plasticizing effect appeared to reach saturation, and excess water may have increasingly acted as a structure-disrupting component. Water molecules can compete with protein side chains for hydrogen bonding, thereby weakening interprotein associations and compromising the stability of the extrusion-induced gel network. Consequently, increasing moisture content from 55% to 70% significantly (*p* < 0.05) reduced the integrity index, which was consistent with the loss of fibrous gel network structure observed in Figure 5.

Barrel temperature was also associated with differences in the integrity index of Y-MA. At a fixed moisture content of 55% and screw speed of 150 rpm, the integrity index was significantly lower at 140 °C than at 150–170 °C, whereas no significant differences were observed among 150, 160, and 170 °C. This result indicates that the sample produced at 140 °C was less resistant to the standardized thermal and mechanical disruption procedure than those produced at higher temperatures. The absence of significant differences among 150–170 °C suggests that further increases in barrel temperature did not measurably improve assay-defined structural integrity under the tested conditions. Previous studies have suggested that increased thermal input may promote protein unfolding, aggregation, and matrix restructuring during high-moisture extrusion [18]. Accordingly, the lower integrity index at 140 °C may reflect insufficient thermal development of the matrix compared with that at 150–170 °C. In contrast, screw speed did not significantly affect the integrity index across the tested range of 150–300 rpm, indicating that shear intensity and mixing conditions within this range had a comparatively limited influence on assay-defined structural retention.

The integrity index values obtained in this study were comparable to those reported in previous studies on HMMA. Using the same or a comparable assay protocol, the integrity index values obtained in the present study ranged from approximately 62% to 92%, partially overlapping with the approximately 45–85% reported by Samard and Ryu [44]. Similarly, Zhang and Ryu [18] observed a significant decrease in integrity index with increasing moisture content in pea protein-based analogs, which is consistent with the present findings. Overall, these results suggest that moisture content strongly influences the stability of the protein-based gel matrix. In the present brewer’s yeast-enhanced system, the decrease in integrity index at higher moisture levels was likely governed mainly by water-mediated weakening of the base protein network rather than by the minor yeast component alone. However, the present integrity-index results do not directly confirm protein unfolding, aggregation, or crosslinking at the molecular level, since the rheological, FTIR, and Raman analyses in this study were not conducted on the extrudates obtained under each processing condition.

### 2.7. Nitrogen Solubility Index (NSI)

**Figure 7:**
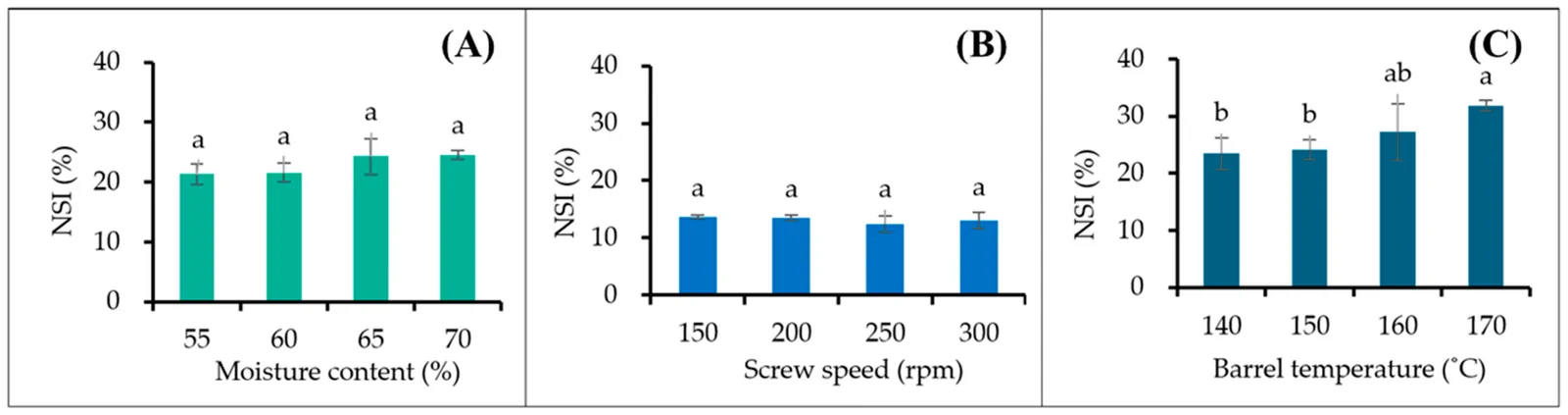
0.5% KOH-extractable nitrogen index of Y-MA produced under different high-moisture extrusion conditions: (**A**) moisture content, (**B**) screw speed, and (**C**) barrel temperature. Data are presented as mean ± SD (n = 3). Different lowercase letters indicate significant differences among treatment levels within each processing parameter (*p* < 0.05).

Figure 7 shows the effects of the individual processing parameters on the nitrogen solubility index (NSI) of Y-MA. NSI indicates the KOH-extractable, ninhydrin-reactive fraction under the assay conditions and may provide indirect information regarding changes in protein extractability during extrusion. A decrease in NSI may be associated with increased aggregation, intermolecular association, or insolubilization, whereas an increase may reflect greater protein extractability resulting from changes in protein conformation, dissociation, or fragmentation.

Changes in this value may be associated with extrusion-induced alterations in protein extractability, including protein unfolding, intermolecular interactions, aggregation, and network formation [47]. Although NSI showed a slight numerical increase as moisture content increased from 55% to 60%, no significant differences were observed among the tested moisture levels. In particular, NSI remained essentially stable between 60% and 70% moisture content, indicating that the extractable nitrogen fraction did not increase further within this higher moisture range. Increased moisture may reduce melt viscosity and thermomechanical processing intensity, which can affect protein extractability during extrusion [18,47]. Within the tested range, moisture content had a limited effect on assay-defined nitrogen extractability, despite its pronounced effects on the integrity index and mechanical properties. The absence of significant NSI differences despite pronounced changes in structural retention and mechanical properties indicates that nitrogen extractability and matrix strength reflect different aspects of the extrudates.

Barrel temperature showed a clearer association with NSI, with an increase observed as the temperature rose from 140 to 170 °C. This trend may indicate changes in the extractability of nitrogen-containing components under the applied assay conditions [20,21]. One possible explanation is that thermal treatment altered protein conformation and intermolecular associations. At lower moisture contents, reduced water plasticization can increase the viscosity of protein-rich melts and alter the shear and deformation conditions experienced during high-moisture extrusion [48]. These rheological conditions may facilitate protein restructuring, although the resulting aggregation pathways depend on protein composition, hydration, and processing history. Previous studies have shown that extrusion-induced network formation in plant protein systems can involve disulfide bonding and non-covalent interactions, including hydrophobic interactions [47,49]. Therefore, the observed NSI trend may reflect temperature-dependent changes in the extractability of nitrogen-containing components under the fixed moisture condition.

The increase in NSI at a higher barrel temperature may reflect temperature-dependent changes in protein extractability. Previous studies have reported that severe thermal treatment during extrusion can reduce protein solubility through aggregation and the formation of less-soluble protein assemblies. Li and Lee [50] observed a marked reduction in wheat protein solubility following extrusion at 160–185 °C, while Nor Afizah and Rizvi [23] reported lower solubility of whey protein concentrate processed at a higher extrusion temperature and attributed this change to increased protein aggregation. More recently, Xia et al. [51] found that increasing the processing temperature of yeast protein isolate-based meat analogs from 160 to 180 °C increased sulfhydryl-group exposure and disulfide-bond formation, suggesting enhanced intermolecular association at greater thermal severity. Taken together, these findings suggest that further increases in barrel temperature beyond the range tested here may shift the dominant mechanism from protein unfolding toward aggregation and reduced solubility. Although the cited studies partially overlap with the present temperature range, their higher upper limits (up to 185 °C) suggest that aggregation may become increasingly dominant at more severe thermal conditions.

In contrast, screw speed did not markedly affect NSI, which remained stable across the tested range. This result suggests that the mechanical shear generated within the tested screw speed range had limited influence on the extractable nitrogen fraction, or that shear-induced changes were more strongly reflected in mechanical properties than in protein solubility. Overall, within the tested high-moisture extrusion framework, barrel temperature showed the clearest association with NSI variation, whereas moisture content had a minor effect and screw speed was the least influential factor.

### 2.8. Mechanical Properties of the Fibrous Protein Gel Matrix

The mechanical properties of Y-MA produced under different extrusion operating parameters are shown in Table 1. Moisture content produced pronounced changes in texture-profile properties and cutting strength. Increasing moisture content significantly decreased springiness, cohesiveness, chewiness, and cutting strength in both directions relative to the fiber orientation. The reduction was particularly evident for chewiness, which decreased by approximately 86% between 55% and 70% moisture content. These results indicate that higher moisture content produced softer, less cohesive extrudates with lower resistance to compression and cutting. Increased water-mediated plasticization and reduced melt viscosity may have contributed to this response.

Barrel temperature showed a generally positive association with the mechanical firmness of Y-MA. Springiness, cohesiveness, chewiness, and cutting strength tended to increase with increasing barrel temperature, although the magnitude and statistical significance of the changes differed among the measured properties. The increases in chewiness and cutting strength indicate that greater thermal input was associated with greater resistance to deformation and fracture under the tested conditions.

Screw speed had a comparatively limited effect on springiness and cohesiveness, which did not differ significantly across the tested range. In contrast, chewiness decreased significantly by approximately 44.8% as screw speed increased from 150 to 300 rpm, and cutting strength generally showed a similar decreasing tendency. Maung et al. [52] reported screw-speed-dependent changes in specific mechanical energy and textural properties of HMMA, while Ribeiro et al. [53] similarly observed greater hardness in soy-based high-moisture extrudates produced at lower screw speeds. The greater mechanical firmness observed at lower screw speed may be associated with a longer residence time and differences in thermomechanical restructuring.

Within the respective tested ranges and fixed processing conditions, substantial changes in the mechanical properties of Y-MA were observed across the moisture-content treatments, consistent with the moisture-dependent changes reported by Zhang and Ryu [18]. These moisture-dependent changes may be related to the strong plasticizing effect of water on melt viscosity and material mobility.

Overall, substantial changes in several mechanical properties were observed across the moisture-content treatments, while barrel temperature and screw speed were also associated with changes in specific textural attributes under their respective fixed processing conditions. The mechanical behavior was closely associated with the macro-fibrous structure shown in Figure 5. Extrudates produced at 55% moisture content exhibited the highest chewiness and cutting strength, corresponding to the dense, longitudinal fibrous structure observed in Figure 5. Conversely, increasing moisture content to 70% resulted in a substantial loss of fiber orientation and a paste-like structure, accompanied by a marked reduction in chewiness. This transition suggests that excessive water over-plasticized the protein melt, reduced melt viscosity, and weakened the stress conditions required for protein alignment and fibrous gel network formation.

The mechanical changes observed in Table 1 can be attributed to differences in protein network formation and phase separation during extrusion. Lower moisture content favored protein–protein interactions by limiting competition from water molecules, resulting in a dense and cohesive gel network with high chewiness and cutting strength. In contrast, excess moisture diluted the protein phase and competed with protein side chains for hydrogen bonding, leading to network weakening and an 86% reduction in chewiness. Higher barrel temperature was associated with greater chewiness and cutting strength under the tested conditions. These changes could be related to temperature-dependent protein restructuring and intermolecular interactions. The texturization degree, calculated as the ratio of vertical to parallel cutting strength (FV/FP), was used to describe directional differences in mechanical resistance. A cutting-strength-based texturization index has similarly been applied to HMMA containing yeast protein isolate [54]. In the present study, the texturization degree varied non-monotonically with moisture content, increased numerically with barrel temperature, and remained within a comparatively narrow range across screw speeds. Thus, the processing conditions substantially influenced overall cutting resistance, whereas their effects on directional mechanical differentiation were less consistent.

The chewiness values obtained in the present study ranged from 866.64 to 6755.82 g and were broadly comparable to those reported for other extruded protein-based meat analogs. Zhang and Ryu [18] reported chewiness values ranging from 1705.61 to 5841.75 g for isolated pea protein-based HMMA under different extrusion conditions. Samard and Ryu [44] reported a chewiness value of 2.96 kg, corresponding to approximately 2960 g, for an intermediate-moisture texturized vegetable protein. Accordingly, chewiness values above 6000 g in the present study were higher than most values reported in the cited pea protein-based study, whereas the lowest value, approximately 867 g at 70% moisture content, reflected a markedly softer and less mechanically resistant matrix. Previous studies suggest that yeast-derived ingredients may influence the fibrous and textural properties of HMMA depending on their composition and inclusion level [10,51,54].

Because the present study employed a sequential one-factor-at-a-time (OFAT) design, interaction effects among moisture content, barrel temperature, and screw speed were neither evaluated nor interpreted. Therefore, the favorable processing conditions identified for each individual factor should not be interpreted as an optimized combined processing condition. Future studies employing factorial experimental designs or response surface methodology (RSM) are needed to evaluate interactions among processing variables.

## 3. Conclusions

This study evaluated the stage-specific effects of moisture content, barrel temperature, and screw speed on the macroscopic fibrous appearance, relative matrix retention, and mechanical properties of brewer’s yeast-enhanced meat analogs (Y-MA). Under the fixed conditions applied at each stage, moisture content was associated with marked changes in matrix retention, chewiness, and cutting strength, while barrel temperature and screw speed affected specific structural and mechanical attributes. Lower moisture content was associated with greater matrix retention under the specified integrity-index test, as well as higher chewiness and cutting strength, whereas increasing moisture content resulted in softer and less mechanically resistant extrudates. Increasing barrel temperature altered the macroscopic fibrous appearance and mechanical firmness, while higher screw speed was generally associated with reduced chewiness and mechanical strength under the tested conditions.

Analyses of the pre-extrusion blends showed that brewer’s yeast altered the pasting and thermal viscoelastic behavior of the protein-starch matrix. Although both blends maintained predominantly elastic behavior, the 10% yeast blend exhibited lower terminal G′ and G″ values after cooling, suggesting reduced network consolidation. In contrast, FTIR and Raman spectra showed no additional resolved bands or appreciable peak shifts, indicating that the rheological changes occurred without major spectroscopically detectable changes in the bulk molecular profile. These findings may reflect altered hydration or physical matrix organization rather than the formation of new chemical structures; however, the underlying mechanism could not be confirmed. Overall, the results indicate that individual extrusion parameters influence the macroscopic and mechanical characteristics of the brewer’s yeast-enhanced formulation under the tested conditions.

Several limitations should be acknowledged. The amino acid composition of the pre-extrusion blends and the resulting extrudates was not analyzed, which limited direct verification of compositional changes associated with brewer’s yeast incorporation and processing. The photographs provided only qualitative morphological information because quantitative image segmentation and image-based fiber-orientation analysis were not conducted. Process-response parameters, including specific mechanical energy, die pressure, torque, and residence time, were not recorded, which limited the evaluation of differences in thermomechanical history among the extrusion treatments. The present integrity-index results do not directly confirm protein unfolding, aggregation, or crosslinking at the molecular level, since the rheological, FTIR, and Raman analyses in this study were not conducted on the extrudates obtained under each processing condition. Sensory or consumer evaluations were not conducted, precluding direct assessment of the relevance of the integrity index to product acceptance. Finally, the one-factor-at-a-time design did not permit evaluation of interactions among moisture content, barrel temperature, and screw speed, and the individually favorable factor levels should not be interpreted as an optimized combined processing condition.

Future studies using factorial designs or response surface methodology, with direct microstructural, rheological, and protein chemistry characterization, are needed to clarify the structural mechanisms and optimize multivariable processing conditions. Recording process-response parameters, including specific mechanical energy, die pressure, torque, and residence time, would enable comparison of the thermomechanical history generated under different extrusion conditions. Amino acid analysis of the blends and extrudates, together with sensory or consumer evaluation, is recommended to establish the compositional and perceptual relevance of the observed structural and mechanical changes.

## 4. Materials and Methods

### 4.1. Materials

Isolated soybean protein (ISP), wheat gluten (WG), and corn starch (CS) were obtained from Pingdingshan Tianjin Plant Albumen Co., Ltd., Pingdingshan, China, Roquette Freres, Lestrem, France, and Samyang Corp., Ulsan, Republic of Korea, respectively.

A base mixture of isolated soybean protein (ISP), wheat gluten (WG), and corn starch (CS) was first prepared at a ratio of 5:4:1 (*w*/*w*, dry basis), corresponding to 50%, 40%, and 10% of the base mixture, respectively. This base mixture constituted 90% of the total dry formulation, and brewer’s yeast (procured from Mungyeong, Republic of Korea) was incorporated at 10% of the total formulation. The brewer’s yeast content was fixed at 10% based on a previous study using a comparable ISP-WG-CS base formulation, which identified 10% brewer’s spent yeast as an appropriate level for texturization [10]. Accordingly, the final dry formulation consisted of 45% ISP, 36% WG, 9% CS, and 10% brewer’s yeast (*w*/*w*).

The crude protein content of ISP, WG, and brewer’s yeast was determined using the AOAC method [55] to be 84.87 ± 0.1%, 79.80 ± 0.1%, and 47.30 ± 0.3%, respectively. The brewer’s yeast contained 2.40 ± 0.10% crude fat, 7.30 ± 0.30% ash, and 9.10 ± 0.30% moisture.

### 4.2. Pasting Properties of Pre-Extrusion Blends

The pasting properties of the pre-extrusion blends containing 0% and 10% brewer’s yeast were determined using a Rapid Visco Analyzer 4800 (PerkinElmer, Waltham, MA, USA), following the procedure described by Flory et al. [56] with minor modifications. The sample amount was adjusted according to the measured moisture content of each blend to obtain an equivalent dry sample weight of 5.5 g. Distilled water was then added to achieve a total sample weight of 30.5 g. The suspensions were subjected to a heating and cooling cycle from 50 to 95 °C and subsequently cooled to 50 °C at a constant paddle speed of 160 rpm. Peak viscosity, trough viscosity, breakdown, final viscosity, setback, peak time, and pasting temperature were automatically calculated from the resulting RVA profiles.

### 4.3. Rheological Properties of Pre-Extrusion Blends

The pre-extrusion blend powder containing 0% or 10% brewer’s yeast (20 g) was dispersed in 100 mL of distilled water and mixed using an overhead stirrer (OSC-20L, JOANLAB, Huzhou, China) at 300 rpm for 1 h. The rheological properties of the prepared dispersions were evaluated according to the method described by Choi et al. [57], using a rotational rheometer (MCR 302e, Anton Paar, Graz, Austria) equipped with a 50 mm parallel-plate geometry. Temperature sweep measurements were conducted at a frequency of 1 Hz and a strain of 0.5%. The samples were heated from 25 °C to 95 °C at a rate of 5 °C/min, held at 95 °C for 5 min, and then cooled back to 25 °C at the same rate. The storage modulus (G′) and loss modulus (G″) were continuously recorded throughout the temperature sweep.

### 4.4. Fourier Transform Infrared Spectroscopy (FTIR)

FTIR was performed to characterize the molecular features of the pre-extrusion blends containing 0% and 10% brewer’s yeast. Spectra were obtained using an FTIR spectrometer (Lyza 7000, Anton Paar, Graz, Austria) according to the method described by Jiang et al. [58]. The spectra were recorded over the wavenumber range of 4000–400 cm^−1^ at a spectral resolution of 4 cm^−1^ using 24 scans per measurement.

### 4.5. Raman Spectroscopy

Raman spectra of the pre-extrusion blends containing 0% and 10% brewer’s yeast were obtained using a Raman spectrometer (Cora 5001 Direct, Anton Paar, Graz, Austria), following the procedure described by Ur-Mora et al. [59] with modifications. Measurements were performed using a 1064 nm excitation laser at a laser power of 150 mW and an exposure time of 10 s. The spectra were collected over the range of 300–1800 cm^−1^. Background subtraction and baseline correction were applied before spectral analysis.

### 4.6. Extrusion

**Figure 8:**
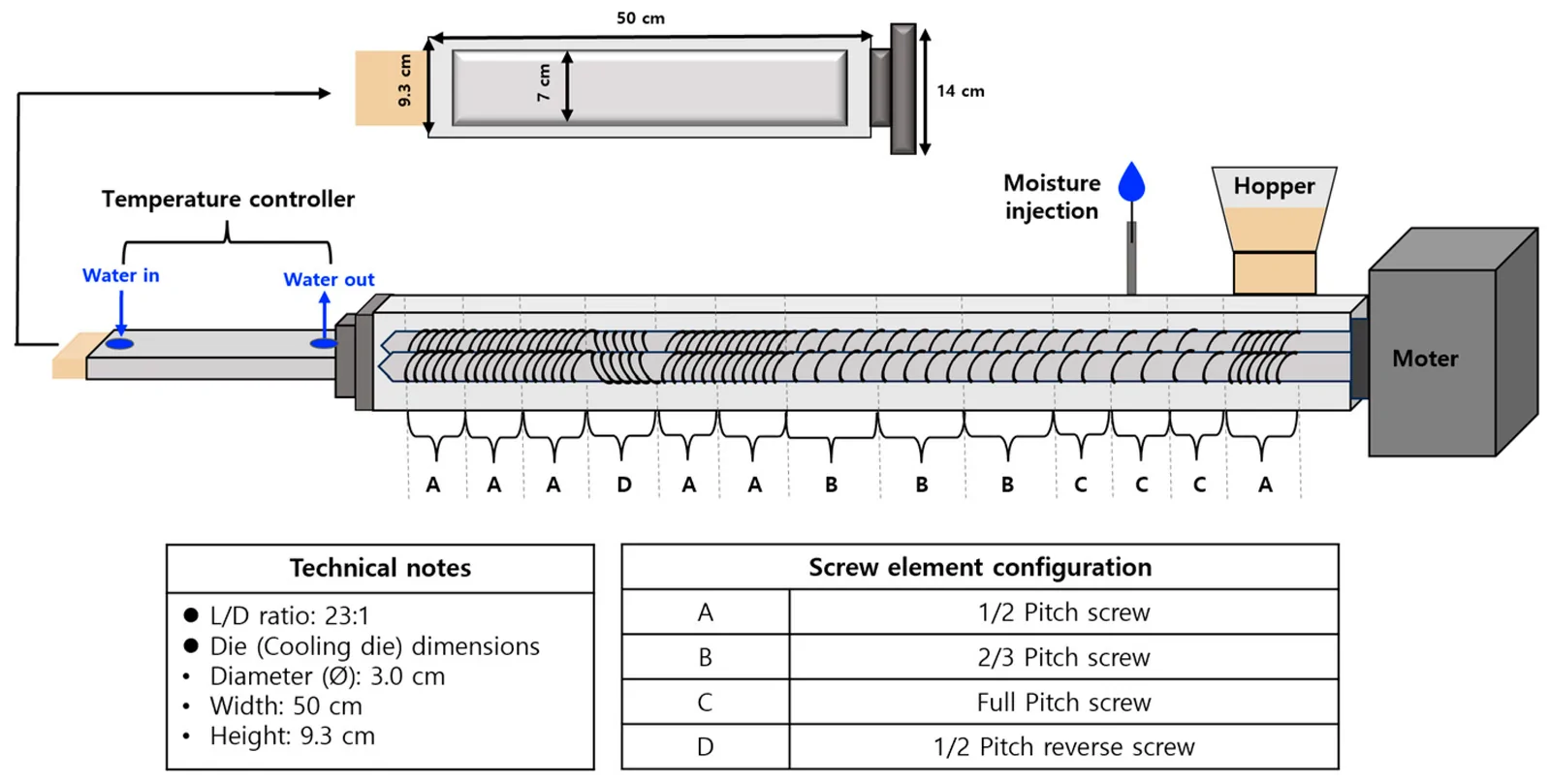
Experimental setup for high-moisture extrusion with screw configuration, redrawn with reference to Jung et al. [60].

Brewer’s yeast-enhanced Y-MA were prepared as structured fibrous protein gel matrices using a co-rotating, intermeshing twin-screw extruder (THK31T, Incheon Machinery Co., Ltd., Incheon, Republic of Korea). The screw configuration (Figure 8) consisted of a 3 cm diameter screw with an L/D ratio of 23:1. During high-moisture extrusion, a slit die (7 × 1 × 50 cm) was used, and its temperature was controlled at 20 °C by circulating water via a temperature control system (Duksan Cotran Co., Daegu, Republic of Korea). Operational parameters during extrusion are outlined in Table 2, with a feeding rate of 100 g/min. A sequential single-factor approach (One-Factor-at-a-Time, OFAT) was employed to evaluate the effects of moisture content, barrel temperature, and screw speed. The fixed parameters for each stage were selected based on preliminary trials, literature references, and extrusion feasibility. Specifically, moisture content was first evaluated (55–70%); subsequently, barrel temperature was tested (140–170 °C) with moisture fixed at 55%. Finally, screw speed (150–300 rpm) was evaluated with moisture and temperature fixed at 55% and 170 °C. Each processing condition was independently evaluated in triplicate. Baseline fixed conditions were selected based on preliminary trials, literature references, and stable extrusion feasibility (55% moisture content, 160–170 °C barrel temperature, and 150 rpm screw speed). Samples were collected 5 min after stable extrusion conditions had been achieved, and all extrudate produced prior to stabilization was discarded. Following extrusion, Y-MA samples were cut into 1 × 1 × 1 cm cubes and stored at 4 °C until analysis of gel network integrity and mechanical properties. For NSI evaluation, selected Y-MA samples underwent freeze-drying, followed by grinding and fractionation through 50- and 70-mesh sieves.

### 4.7. Integrity Index

The integrity index was used to evaluate the stability of the structured protein gel matrix against thermal treatment, homogenization, and washing. It was assessed using a method adapted from Samard and Ryu [44]. Samples (1 × 1 × 1 cm, 5 g, dry basis) were combined with 100 mL of distilled water and processed in an autoclave at 121 °C for 15 min (PAC-60, Lab House Co., Ltd., Seoul, Republic of Korea). Subsequently, the samples were passed through a 20-mesh screen, then rapidly cooled under running tap water for 1 min before being transferred to a 200 mL beaker containing 100 mL of distilled water. Homogenization was carried out for 1 min at a speed of 17,450 rpm using an IKA disperser (T10 Basic Ultra-Turrax, IKA Co., Ltd., Seoul, Republic of Korea). The samples underwent a second sieving using a 20-mesh screen, followed by rinsing with tap water for 1 min and drying at 105 °C until a stable weight was achieved. Measurements were performed in triplicate, and the results were averaged. This protocol was used as a standardized severe hydrothermal and mechanical challenge test for comparing the relative structural resistance of the samples. The integrity index was calculated according to Equation (1).

(1) $$\mathrm{Integrity}\;\mathrm{index}\;\left(\%\right)=\frac{W_{r}}{W_{i}}\times 100$$

where $W_{r}$ is the dry weight of the retained residue and $W_{i}$ is the initial dry weight of the sample.

### 4.8. Nitrogen Solubility Index

The nitrogen solubility index (NSI) was determined as a comparative indicator of changes in the 0.5% KOH-extractable, ninhydrin-reactive nitrogen fraction following extrusion. NSI was determined with slight modifications based on previously reported methods used for extruded protein products and meat analogs [44,61]. Ground samples (0.1 g) were dispersed in 5 mL of 0.5% KOH and shaken at 200 rpm for 30 min using a shaker (SI-300R, Jeio Tech Co., Daejeon, Republic of Korea). The mixture was then centrifuged at 3000 rpm for 30 min, and the supernatant was collected as Solution A. For the preparation of the acid-hydrolyzed reference fraction, 0.1 g of the ground sample was mixed with 2.5 mL of HCl and hydrolyzed at 100 °C for 24 h. After hydrolysis, 5 mL of distilled water was added, and the mixture was centrifuged to obtain a clear supernatant designated as Solution B. The ninhydrin-reactive amino-group contents of Solutions A and B were subsequently determined using the ninhydrin method [62]. NSI measurements were performed in triplicate, and the results are presented as mean values. NSI was calculated according to Equation (2).

(2) $$\mathrm{Nitrogen}\;\mathrm{solubility}\;\mathrm{index}\;\left(\%\right)=\frac{N_{e}}{N_{h}}\;\times 100$$

where $N_{e}$ is the amount of ninhydrin-reactive amino-group equivalents measured in the 0.5% KOH-extractable fraction (Solution A), and $N_{h}$ is the corresponding amount in the acid-hydrolyzed reference fraction (Solution B). The extraction volumes and dilution factors were taken into account in calculating both values.

### 4.9. Mechanical Characterization of the Fibrous Protein Gel Matrix

To assess the mechanical properties of the fibrous protein gel matrix, texture profile analysis (TPA) and cutting strength measurements of Y-MA were conducted using a modified version of a previously published method [63]. For TPA measurements, Y-MA cubes (1 × 1 × 1 cm) were compressed with a cylindrical probe (25 mm diameter) using a Sun rheometer (Compac-100, Sun Science Corporation, Tokyo, Japan). Springiness, cohesiveness, and chewiness were measured with the maximum load set to 10 kg and a 20 mm gap between the grips. Samples were compressed to 50% of their original height at a crosshead speed of 100 mm/min. For TPA measurements, the samples were positioned with the extrusion-induced fiber orientation parallel to the instrument platform and perpendicular to the compression direction. Thus, compression was applied across the fiber orientation, and the same sample orientation was maintained for all treatments. Cutting strength measurements were performed on Y-MA samples (1 × 1 × 1.5 cm) in both vertical (FV) and parallel (FP) orientations using a blade probe (7.5 mm × 38.3 mm; Sun Science Corporation, Tokyo, Japan), with the maximum load set to 10 kg. The texturization degree was calculated for each replicate by dividing the cutting strength measured in the vertical direction by that measured in the parallel direction (FV/FP). Springiness, cohesiveness, chewiness, cutting strength, and texturization degree were determined using equations from Trinh and Glasgow [64], as indicated in Equations (3)–(7). For each independent extrusion run, three samples were measured and averaged before statistical analysis. Results are expressed as mean ± standard deviation of three independent extrusion runs (n = 3).

(3) $$\mathrm{Springiness}\;\left(\%\right)=\frac{D_{2}}{D_{1}}\;\times 100$$

where *D*_1_ denotes the original compression distance, while *D*_2_ indicates the measured height in the second compression cycle.

(4) $$\mathrm{Cohesiveness}\;\left(\%\right)=\frac{A_{2}}{A_{1}}\;\times 100$$

where *A*_1_ and *A*_2_ are the areas under the force-deformation curves during the first and second compressions, respectively.

(5) $$\mathrm{Chewiness}\;\left(g\right)=\frac{\mathrm{Springiness}}{100}\times \;\frac{\mathrm{Cohesiveness}}{100}\;\times \mathrm{Maximum}\;\mathrm{stress}$$

(6) $$\mathrm{Cutting}\;\mathrm{strength}\;({g/\mathrm{cm}}^{2})=\frac{\mathrm{Maximum}\;\mathrm{stress}\;(g)}{\mathrm{Cross}\;\mathrm{sectional}\;\mathrm{area}\;({\mathrm{cm}}^{2})}$$

(7) $$\mathrm{Texturization}\;\mathrm{degree}=\frac{F_{V}}{F_{P}}$$

where $F_{V}$ and $F_{P}$ represent the cutting strengths measured in the vertical and parallel directions relative to the fiber orientation, respectively.

The rheometer was operated under manufacturer-recommended standard settings, and all measurements were performed under consistent sample dimensions and test conditions.

### 4.10. Statistical Analysis

Statistical analysis was conducted using SPSS software (version 27.0; IBM Corp., Armonk, NY, USA). For the sequential one-factor-at-a-time (OFAT) experiment, separate one-way analyses of variance (ANOVAs) were conducted for each processing factor (moisture content, barrel temperature, and screw speed). The independent extrusion run was treated as the experimental unit (n = 3). Normality of residuals and homogeneity of variance were evaluated using the Shapiro–Wilk test and Levene’s test, respectively. When a significant effect was detected by ANOVA, differences among treatment means were assessed using Duncan’s multiple range test at a significance level of *p* < 0.05. The same statistical approach was applied to all response variables analyzed for treatment comparisons.

## Figures and Tables

**Table 1:** Mechanical properties of fibrous protein gel matrices in Y-MA produced under different high-moisture extrusion conditions.

Extrusion Process Parameters			Springiness (%)	Cohesiveness (%)	Chewiness (g)	Cutting Strength (g/cm^2^)		Texturization Degree
MC (%)	BT (°C)	SS (rpm)				Vertical Direction	Parallel Direction	
55	160	150	92.39 ± 0.5 ^b^	77.45 ± 2.2 ^c^	6211.09 ± 392.1 ^b^	2070.97 ± 214.9 ^a^	1524.17 ± 236.43 ^a^	1.37 ± 0.1 ^bc^
60			91.25 ± 1.0 ^c^	72.00 ± 2.3 ^d^	3544.28 ± 334.7 ^g^	1107.61 ± 57.4 ^f^	1062.86 ± 97.0 ^de^	1.05 ± 0.1 ^d^
65			89.75 ± 0.7 ^de^	68.72 ± 2.1 ^e^	1390.95 ± 154.4 ^h^	768.44 ± 80.6 ^g^	676.60 ± 46.1 ^g^	1.13 ± 0.1 ^d^
70			89.33 ± 1.2 ^e^	64.52 ± 1.6 ^f^	866.64 ± 85.6 ^i^	533.42 ± 56.8 ^h^	423.19 ± 24.8 ^h^	1.26 ± 0.1 ^c^
55	140	150	90.24 ± 0.7 ^d^	73.45 ± 5.8 ^d^	4493.61 ± 915.8 ^f^	1427.30 ± 137.4 ^e^	1294.54 ± 63.7 ^c^	1.11 ± 0.1 ^d^
	150		92.05 ± 1.1 ^bc^	78.92 ± 0.6 ^bc^	4770.10 ± 243.1 ^ef^	1645.69 ± 162.7 ^d^	1306.59 ± 180.8 ^bc^	1.29 ± 0.2 ^c^
	160		92.05 ± 0.5 ^bc^	78.96 ± 1.6 ^bc^	5336.45 ± 297.1 ^cd^	1754.67 ± 153.4 ^cd^	1342.16 ± 162.7 ^bc^	1.31 ± 0.1 ^bc^
	170		92.51 ± 0.6 ^b^	79.15 ± 0.6 ^abc^	5407.04 ± 199.1 ^cd^	1955.38 ± 269.2 ^ab^	1485.61 ± 386.9 ^ab^	1.35 ± 0.2 ^bc^
55	170	150	94.01 ± 0.8 ^a^	81.53 ± 1.0 ^a^	6755.82 ± 277.9 ^a^	1822.78 ± 110.4 ^bc^	1219.36 ± 168.4 ^cd^	1.51 ± 0.1 ^a^
		200	94.05 ± 0.7 ^a^	81.26 ± 0.9 ^ab^	5578.54 ± 342.4 ^c^	1722.21 ± 151.4 ^cd^	1201.55 ± 133.4 ^cd^	1.43 ± 0.5 ^ab^
		250	94.85 ± 0.6 ^a^	81.59 ± 0.9 ^a^	5055.24 ± 274.0 ^de^	1469.69 ± 163.1 ^e^	960.86 ± 93.5 ^ef^	1.53 ± 0.1 ^a^
		300	94.52 ± 0.6 ^a^	80.83 ± 1.4 ^ab^	3730.37 ± 103.2 ^g^	1318.63 ± 105.1 ^e^	854.44 ± 128.0 ^f^	1.56 ± 0.1 ^a^

Data are presented as mean ± SD of three independent extrusion runs (n = 3). Different superscript letters within the same column indicate significant differences (*p* < 0.05) according to Duncan’s multiple range test. MC (moisture content), BT (barrel temperature), and SS (screw speed). The coefficients of variation (CVs) for the measured variables ranged from 0.5% to 26.0% across all treatments.

**Table 2:** Extrusion operation parameters for high-moisture extrusion cooking of Y-MA.

Extrusion Operation Parameters		
Moisture Content (%)	Barrel Temperature (°C)	Screw Speed (rpm)
55	Fixed at 160	Fixed at 150
60		
65		
70		
Fixed at 55	140	Fixed at 150
	150	
	160	
	170	
Fixed at 55	Fixed at 170	150
		200
		250
		300

## Data Availability

The original contributions presented in this study are included in the article. Further inquiries can be directed to the corresponding author.

## Abbreviations

The following abbreviations are used in this manuscript:

HMMA	High-moisture meat analog(s)
Y-MA	Yeast-enhanced meat analog(s)
MC	Moisture content
BT	Barrel temperature
SS	Screw speed
NSI	Nitrogen solubility index
TPA	Texture profile analysis
RVA	Rapid Visco Analyzer
FTIR	Fourier transform infrared spectroscopy
ISP	Isolated soybean protein
WG	Wheat gluten
CS	Corn starch
OFAT	One-factor-at-a-time
RSM	Response surface methodology
CV(s)	Coefficient(s) of variation
FV	Cutting strength, vertical direction
FP	Cutting strength, parallel direction

## References

[1] Gulzar S., Hosseini A.F., Martín-Belloso O., Soliva-Fortuny R., Rizvi S.S. (2025). Engineering Processes for Plant-Based Meat Analogs: Current Status and Future Outlook. Compr. Rev. Food Sci. Food Saf..

[2] Benković M., Jurinjak Tušek A., Sokač Cvetnić T., Jurina T., Valinger D., Gajdoš Kljusurić J. (2023). An Overview of Ingredients Used for Plant-Based Meat Analogue Production and Their Influence on Structural and Textural Properties of the Final Product. Gels.

[3] Jang J., Lee D.W. (2024). Advancements in Plant-Based Meat Analogs Enhancing Sensory and Nutritional Attributes. NPJ Sci. Food.

[4] Sha L., Xiong Y.L. (2020). Plant Protein-Based Alternatives of Reconstructed Meat: Science, Technology, and Challenges. Trends Food Sci. Technol..

[5] Agboola J.O., Lapeña D., Øverland M., Arntzen M.Ø., Mydland L.T., Hansen J.Ø. (2022). Yeast as a novel protein source—Effect of species and autolysis on protein and amino acid digestibility in Atlantic salmon (Salmo salar). Aquaculture.

[6] Podpora B., Świderski F., Sadowska A., Rakowska R., Wasiak-Zys G. (2016). Spent brewer’s yeast extracts as a new component of functional food. Czech. J. Food Sci..

[7] Zeko-Pivač A., Habschied K., Kulisic B., Barkow I., Tišma M. (2023). Valorization of Spent Brewer’s Yeast for the Production of High-Value Products, Materials, and Biofuels and Environmental Application. Fermentation.

[8] Łukaszewicz M., Leszczyński P., Jabłoński S.J., Kawa-Rygielska J. (2024). Potential Applications of Yeast Biomass Derived from Small-Scale Breweries. Appl. Sci..

[9] Puligundla P., Mok C., Park S. (2020). Advances in the valorization of spent brewer’s yeast. Innov. Food Sci. Emerg. Technol..

[10] Jeon Y.-H., Gu B.-J., Ryu G.-H. (2022). Effects of Brewer’s Spent Yeast Content on the Physicochemical Properties of Extruded High-Moisture Meat Analog. J. Korean Soc. Food Sci. Nutr..

[11] Pinho L.S., Brexó R.P., Costa T.d.J., Thomazini M., Campanella O.H., Favaro-Trindade C.S. (2025). Production of Vitamin D3-Fortified Plant-Based Meat Analogs Through High-Moisture Extrusion. Foods.

[12] Zahari I., Östbring K., Purhagen J.K., Rayner M. (2022). Plant-Based Meat Analogues from Alternative Protein: A Systematic Literature Review. Foods.

[13] Chen Q., Zhang J., Zhang Y., Meng S., Wang Q. (2021). Rheological properties of pea protein isolate-amylose/amylopectin mixtures and the application in the high-moisture extruded meat substitutes. Food Hydrocoll..

[14] Pietsch V.L., Werner R., Karbstein H.P., Emin M.A. (2019). High moisture extrusion of wheat gluten: Relationship between process parameters, protein polymerization, and final product characteristics. J. Food Eng..

[15] Zhang Y., Jin T., Ryu G., Gao Y. (2021). Effects of screw configuration on chemical properties and ginsenosides content of extruded ginseng. Food Sci. Nutr..

[16] Guerrero P., Beatty E., Kerry J.P., de la Caba K. (2012). Extrusion of soy protein with gelatin and sugars at low moisture content. J. Food Eng..

[17] Lin S., Huff H.E., Hsieh F. (2002). Extrusion Process Parameters, Sensory Characteristics, and Structural Properties of a High Moisture Soy Protein Meat Analog. J. Food Sci..

[18] Zhang Y., Ryu G.H. (2023). Effects of Process Variables on the Physicochemical, Textural, and Structural Properties of an Isolated Pea Protein-Based High-Moisture Meat Analog. Foods.

[19] Li R., Wang C., Wang Y., Xie X., Sui W., Liu R., Wu T., Zhang M. (2023). Extrusion Modification of Wheat Bran and Its Effects on Structural and Rheological Properties of Wheat Flour Dough. Foods.

[20] Hossain Brishti F., Chay S.Y., Muhammad K., Rashedi Ismail-Fitry M., Zarei M., Karthikeyan S., Caballero-Briones F., Saari N. (2021). Structural and rheological changes of texturized mung bean protein induced by feed moisture during extrusion. Food Chem..

[21] Choi S.-M., Ma C.-Y. (2005). Conformational Study of Globulin from Common Buckwheat (Fagopyrum esculentum Moench) by Fourier Transform Infrared Spectroscopy and Differential Scanning Calorimetry. J. Agric. Food Chem..

[22] Zhou L., Yang Y., Ren H., Zhao Y., Wang Z., Wu F., Xiao Z. (2016). Structural Changes in Rice Bran Protein upon Different Extrusion Temperatures: A Raman Spectroscopy Study. J. Chem..

[23] Nor Afizah M., Rizvi S.S.H. (2014). Functional properties of whey protein concentrate texturized at acidic pH: Effect of extrusion temperature. LWT.

[24] Zhang Z., Zhang L., He S., Li X., Jin R., Liu Q., Chen S., Sun H. (2023). High-Moisture Extrusion Technology Application in the Processing of Textured Plant Protein Meat Analogues: A Review. Food Rev. Int..

[25] See X.Y., Chiang J.H., Law L.M., Osen R. (2025). High Moisture Extrusion of Plant Proteins: Advances, Challenges, and Opportunities. Crit. Rev. Food Sci. Nutr..

[26] Zhang L., Dong L., Zhang H., He Y., Ma X. (2025). Effects of Yeast β-Glucan on Gelatinization, Structure and Digestibility of Potato Starch. J. Sci. Food Agric..

[27] Teobaldi A.G., Carrillo Parra E.J., Barrera G.N., Ribotta P.D. (2025). Effect of Food Proteins on Wheat Starch Pasting and Thermal Properties. Foods.

[28] Satrapai S., Suphantharika M. (2007). Influence of Spent Brewer’s Yeast β-Glucan on Gelatinization and Retrogradation of Rice Starch. Carbohydr. Polym..

[29] Wang J., Ji Z., Zhao Z., Zhang Y., Guo S., Yan M., Xu K., Guo M. (2025). Food Potential of Wheat Brewer’s Spent Grain Modified by Extrusion: Impact on the Characteristics of Wheat Flour and Commercial Steamed Bread. J. Cereal Sci..

[30] Ignatzy L.M., Kern K., Muranyi I.S., Alpers T., Becker T., Gola S., Schweiggert-Weisz U. (2026). Thermal, Rheological, and Microstructural Characterization of Composite Gels from Fava Bean Protein and Pea Starch. Food Hydrocoll..

[31] Banchathanakij R., Suphantharika M. (2009). Effect of Different β-Glucans on the Gelatinisation and Retrogradation of Rice Starch. Food Chem..

[32] Cui Y., Han X., Huang X., Li S. (2023). Effects of Different Sources of β-Glucan on Pasting, Gelation, and Digestive Properties of Pea Starch. Food Hydrocoll..

[33] Dahl J.F., Bouché O., Corredig M. (2025). Multiscale Study of Structure Formation in High Moisture Extruded Plant Protein Biopolymer Mixes. Food Hydrocoll..

[34] Wójcik M., Różyło R., Schönlechner R., Matwijczuk A., Dziki D. (2022). Low-Carbohydrate, High-Protein, and Gluten-Free Bread Supplemented with Poppy Seed Flour: Physicochemical, Sensory, and Spectroscopic Properties. Molecules.

[35] Ørskov K.E., Bach Christensen L., Wiking L., Hannibal T., Hammershøj M. (2023). Detecting Interactions between Starch and Casein in Imitation Cheese by FTIR and Raman Spectroscopy. Food Chem. Adv..

[36] Nilsson K., Johansson M., Sandström C., Eriksson Röhnisch H., Hedenqvist M.S., Langton M. (2023). Pasting and Gelation of Faba Bean Starch-Protein Mixtures. Food Hydrocoll..

[37] Zhu Y., Li Y., Wu C., Teng F., Qi B., Zhang X., Zhou L., Yu G., Wang H., Zhang S. (2019). Stability Mechanism of Two Soybean Protein-Phosphatidylcholine Nanoemulsion Preparation Methods from a Structural Perspective: A Raman Spectroscopy Analysis. Sci. Rep..

[38] Miyake R., Ando M., Kobayashi K., Nagai K., Ohshiro T., Tomoda H., Takeyama H. (2025). Raman Imaging of an Amphotericin B Potentiator in Single-Cell *Saccharomyces cerevisiae*. J. Phys. Chem. B.

[39] Stawoska I., Wesełucha-Birczyńska A., Skoczowski A., Dziurka M., Waga J. (2021). FT-Raman Spectroscopy as a Tool to Study the Secondary Structures of Wheat Gliadin Proteins. Molecules.

[40] Arêas J.A.G. (1992). Extrusion of food proteins. Crit. Rev. Food Sci. Nutr..

[41] Osen R., Toelstede S., Wild F., Eisner P., Schweiggert-Weisz U. (2014). High moisture extrusion cooking of pea protein isolates: Raw material characteristics, extruder responses, and texture properties. J. Food Eng..

[42] Kuijpers S.A., Goudappel G.J., Huppertz T., van Duynhoven J.P.M., Terenzi C. (2024). Quantification of Phase Separation in High Moisture Soy Protein Extrudates by NMR and MRI. Food Res. Int..

[43] Emin M.A., Quevedo M., Wilhelm M., Karbstein H.P. (2017). Analysis of the reaction behavior of highly concentrated plant proteins in extrusion-like conditions. Innov. Food Sci. Emerg. Technol..

[44] Samard S., Ryu G.-H. (2019). A comparison of physicochemical characteristics, texture, and structure of meat analogue and meats. J. Sci. Food Agric..

[45] Nasrollahzadeh F., Alexi N., Skov K.B., Roman L., Sfyra K., Martinez M.M. (2024). Texture Profiling of Muscle Meat Benchmarks and Plant-Based Analogues: An Instrumental and Sensory Design Approach with Focus on Correlations. Food Hydrocoll..

[46] Knaapila A., Kantanen K., Ramos-Diaz J.M., Piironen V., Sandell M., Jouppila K. (2024). Sensory and Physical Properties of Fibrous Meat Analogs Made from Faba Bean, Pea, and Oat Using High-Moisture Extrusion. Foods.

[47] Liu K., Hsieh F.-H. (2008). Protein-Protein Interactions during High-Moisture Extrusion for Fibrous Meat Analogues and Comparison of Protein Solubility Methods Using Different Solvent Systems. J. Agric. Food Chem..

[48] Purcell T., Delaplace G., Riaublanc A., Demême M., Derensy A., Della Valle G. (2025). A General Viscosity Model for High Moisture Extrudates of Pea Protein Isolates/Gluten Blend. Phys. Fluids.

[49] Gräfenhahn M., Beyrer M. (2026). From Isolate to Concentrate: Insights into the Texturization of Pea Protein Systems during High-Moisture Extrusion. Innov. Food Sci. Emerg. Technol..

[50] Li M., Lee T.-C. (1996). Effect of Extrusion Temperature on Solubility and Molecular Weight Distribution of Wheat Flour Proteins. J. Agric. Food Chem..

[51] Xia S., Song J., Ma C., Hao T., Hou Y., Shen S., Li K., Ma L., Xue Y., Xue C. (2023). Effects of Moisture Content and Processing Temperature on the Strength and Orientation Regulation of Fibrous Structures in Meat Analogues. Food Hydrocoll..

[52] Maung T.-T., Gu B.-Y., Ryu G.-H. (2021). Influence of Extrusion Process Parameters on Specific Mechanical Energy and Physical Properties of High-Moisture Meat Analog. Int. J. Food Eng..

[53] Ribeiro G., Piñero M.-Y., Parle F., Blanco B., Roman L. (2024). Optimizing Screw Speed and Barrel Temperature for Textural and Nutritional Improvement of Soy-Based High-Moisture Extrudates. Foods.

[54] Choi H.W., Hahn J., Choi Y.J. (2026). Protein Network Density and Fibrous Structure Control of Soy Protein Isolate-Based High-Moisture Meat Analogs Using Yeast or Rice Protein Isolates with Distinct Glass Transition Temperatures. Food Hydrocoll..

[55] AOAC International (2005). Official Methods of Analysis of AOAC International.

[56] Flory J., Xiao R., Li Y., Dogan H., Talavera M.J., Alavi S. (2023). Understanding Protein Functionality and Its Impact on Quality of Plant-Based Meat Analogues. Foods.

[57] Choi H.W., Hahn J., Kim H.-S., Choi Y.J. (2026). Impact of Rice Flour with Different Amylose Contents on the Texturization of Plant Protein by High-Moisture Extrusion. Food Hydrocoll..

[58] Jiang L., Zhang H., Zhang J., Liu S., Tian Y., Cheng T., Guo Z., Wang Z. (2024). Improve the Fiber Structure and Texture Properties of Plant-Based Meat Analogues by Adjusting the Ratio of Soy Protein Isolate (SPI) to Wheat Gluten (WG). Food Chem. X.

[59] Ur-Mora D., Hernández-Meléndez O., Bárzana E. (2025). Effects of Pectin and Avocado Oil in the Production of Meat Analogues Obtained by High Moisture Extrusion and Their Physicochemical Characterization. Sustain. Food Technol..

[60] Jung H., Gu B.J., Jung D.E. (2026). Upcycled Apple Pomace as an Innovative Ingredient in High-Moisture Meat Analogs: Sustainable Valorization for Food Production. Sustainability.

[61] Daun J.K., DeClercq D.R. (1994). Comparison of combustion and Kjeldahl methods for determination of nitrogen in oilseeds. J. Am. Oil Chem. Soc..

[62] Starcher B. (2001). A Ninhydrin-Based Assay to Quantitate the Total Protein Content of Tissue Samples. Anal. Biochem..

[63] Samard S., Gu B.Y., Ryu G.H. (2019). Effects of extrusion types, screw speed and addition of wheat gluten on physicochemical characteristics and cooking stability of meat analogues. J. Sci. Food Agric..

[64] Trinh K.T., Glasgow S. On the Texture Profile Analysis Test. Proceedings of the Chemeca 2012: Quality of Life Through Chemical Engineering.

